# Four-year follow-up of a randomized controlled trial of choline for neurodevelopment in fetal alcohol spectrum disorder

**DOI:** 10.1186/s11689-020-09312-7

**Published:** 2020-03-12

**Authors:** Jeffrey R. Wozniak, Birgit A. Fink, Anita J. Fuglestad, Judith K. Eckerle, Christopher J. Boys, Kristin E. Sandness, Joshua P. Radke, Neely C. Miller, Christopher Lindgren, Ann M. Brearley, Steven H. Zeisel, Michael K. Georgieff

**Affiliations:** 1 University of Minnesota Twin Cities, Minneapolis, MN USA; 2 Department of Psychiatry, University of Minnesota, F282 / 2A West, 2450 Riverside Ave, Minneapolis, MN 55454 USA; 3 University of North Florida, Jacksonville, FL USA; 4 Fagron Inc., St. Paul, MN USA; 5 University of North Carolina, Nutrition Research Institute, Kannapolis, NC USA

**Keywords:** Fetal alcohol spectrum disorders, Choline, Cognition, Randomized controlled trials, Longitudinal studies

## Abstract

**Background:**

Despite the high prevalence of fetal alcohol spectrum disorder (FASD), there are few interventions targeting its core neurocognitive and behavioral deficits. FASD is often conceptualized as static and permanent, but interventions that capitalize on brain plasticity and critical developmental windows are emerging. We present a long-term follow-up study evaluating the neurodevelopmental effects of choline supplementation in children with FASD 4 years after an initial efficacy trial.

**Methods:**

The initial study was a randomized, double-blind, placebo-controlled trial of choline vs. placebo in 2–5-year-olds with FASD. Participants include 31 children (16 placebo; 15 choline) seen 4 years after trial completion. The mean age at follow-up was 8.6 years. Diagnoses were 12.9% fetal alcohol syndrome (FAS), 41.9% partial FAS, and 45.1% alcohol-related neurodevelopmental disorder. The follow-up included measures of intelligence, memory, executive functioning, and behavior.

**Results:**

Children who received choline had higher non-verbal intelligence, higher visual-spatial skill, higher working memory ability, better verbal memory, and fewer behavioral symptoms of attention deficit hyperactivity disorder than the placebo group. No differences were seen for verbal intelligence, visual memory, or other executive functions.

**Conclusions:**

These data support choline as a potential neurodevelopmental intervention for FASD and highlight the need for long-term follow-up to capture treatment effects on neurodevelopmental trajectories.

**Trial registration:**

ClinicalTrials.Gov #NCT01149538; Registered: June 23, 2010; first enrollment July 2, 2010

## Introduction

Fetal alcohol spectrum disorders (FASDs) comprise a range of effects resulting from prenatal alcohol exposure (PAE) including neurological abnormalities, cognitive and behavioral impairments, growth retardation, and craniofacial anomalies [1]. FASD affects 0.8% of the world’s population, 2.0 to 5.0% of the European and North American populations [2, 3], and 13.6 to 28% of high-risk rural populations in South Africa [4, 5]. Despite being more common than autism spectrum disorder, which has a prevalence of 0.6% [6], FASD remains under-recognized [7]. Very few treatments have been investigated despite FASD’s tremendous public health burden including cognitive disability, mental health comorbidity, productivity loss, educational and employment challenges, homelessness, addiction, and legal difficulties [8, 9]. Cognitive deficits are a core feature of FASD, ranging from serious intellectual impairment to more select deficits in attention, executive functioning, memory, visual-perceptual/motor skills, and academics [10]. Cognition is a natural target for intervention in FASD because cognitive deficits contribute to problems with adaptive functioning, social skills, and capacity for independent living [11].

One potential intervention for the cognitive impairments associated with FASD is the essential nutrient choline [12] which is known to have a direct impact on brain development and cognition [13]. In pre-clinical models, perinatal choline availability impacts multiple aspects of neurodevelopment, especially in the hippocampus; choline augmentation contributes to increased dendritic arborization in CA1, larger cells, and functional changes to the cells [14–17]. Choline affects the hippocampal cholinergic system and alters brain structure and function in regions essential for memory functioning, including methylation in the hippocampus and prefrontal cortex [13, 18–20]. Prenatal and postnatal choline supplementation also affects choline acetyltransferase levels in the hippocampus and frontal cortex in rats which are associated with improved memory functioning, especially visual-spatial memory [21, 22]. Rodent data demonstrate that the hippocampus and memory processes dependent on it are targets of prenatal alcohol exposure (PAE) [23–25] and that supplementation with choline can reduce the severity of these learning and memory deficits caused by PAE [26, 27]. In addition, a sheep model of prenatal choline supplementation following PAE has demonstrated significant benefits for brain and eye development [28].

Only a handful of human choline studies for individuals with PAE/FASD have been undertaken (Table 1). One study employing prenatal choline supplementation in pregnant Ukrainian women demonstrated improvements in an infant attentional/memory task (cardiac response to familiar/unfamiliar visual stimuli) [31]. That study did not observe benefits for prenatal choline supplementation on global cognitive functioning in the offspring above the benefits from a multi-vitamin supplement [32]. A recently published study found that providing prenatal oral choline supplementation (2 g) to pregnant South African women who were consuming alcohol had a beneficial impact on the development of the offspring (increased catch-up weight and head circumference as well as improvements in an eyeblink-conditioning response and on a visual recognition memory task) [34].

**Table 1:** Existing studies evaluating choline in children with fetal alcohol spectrum disorder

Study	Supplementation type and dose	Age range and duration	*N*	Outcome
Wozniak et al. 2013 [29]	Choline bitartrate; 513 mg choline vs. placebo per day	2 to 5 years old; 9-month duration	10 choline; 9 placebo	Feasibility and tolerability: dose taken on 87% of days with no difference between choline and placebo; fishy odor occurred in 56% (choline) vs. 0% (placebo)
Wozniak et al. 2015* [30]	Choline bitartrate; 513 mg choline vs. placebo per day	2 to 5 years old; 9-month duration	31 choline; 20 placebo	Efficacy: no choline effect for global cognition; Significant choline effect for delayed sequential memory in 2–3-year-olds compared to 4–5-year-olds
Kable et al. 2015 [31]	750 mg choline + multi-vitamin vs. multi-vitamin vs. standard care per day	Prenatal; from first prenatal clinic visit through delivery	37 choline; 50 multi-vitamin; 87 multi-vitamin + treatment; 81 standard care	Efficacy: significant choline effect for cardiac orienting response as a measure of visual attention/memory encoding
Coles et al. 2015 [32]	750 mg choline+multi-vitamin vs. multi-vitamin vs. standard care per day	Prenatal; from first prenatal clinic visit through delivery	95 choline + multi-vitamin; 96 multi-vitamin; 176 standard care	Efficacy: no choline effect for global measure of infant cognition and motor skill.
Nguyen et al. 2016 [33]	Glycerophospho-choline; 625 mg choline per day	5 to 10 years old; 6-week duration	29 choline; 26 placebo	Efficacy: no significant choline effect for measures of paired-associate learning, fluency, working memory, attention, or fine motor speed
Jacobson et al. 2018 [34]	Choline bitartrate; 2000 mg choline vs. placebo per day	Prenatal; varied duration, < 23 weeks delivery	34 choline; 35 placebo	Feasibility and tolerability: minimal side effects consisting of increased nausea/dyspepsia in the choline group vs. placebo.
Jacobson et al. 2018 [34]	Choline bitartrate; 2000 mg choline vs. placebo per day	Prenatal; varied duration, < 23 weeks delivery	34 choline; 35 placebo	Efficacy: significant choline effect for eye-blink conditioning and visual recognition memory
Sarkar et al. 2019 [35]	Choline bitartrate; 500 mg choline vs. placebo per day	2.6 to 5 years; 9-month duration	Varies	Decreased methylation of hPER2 and hPOMC genes and increased expression of hPER2 and hPOMC genes.

*Notes: 19 participants from Wozniak et al. 2013 [29] were included in Wozniak et al. 2015 [30]

*hPER2* human period2 gene, *hPOMC* human pro-opiomelanocortin

Although it is likely that pre-natal choline supplementation will be associated with broader benefits than post-natal supplementation, evidence of post-natal effects is critically needed because some alcohol-exposed pregnancies are only identified retrospectively and also because of the relative dearth of interventions available to affected children. Our early double-blind, randomized, placebo-controlled pilot study established relative safety and tolerability of choline in 20 children with FASD [29]. A subsequent trial by our group including 40 additional children revealed beneficial effects for hippocampus-mediated sequential delayed memory in young (ages 2–3) but not older (ages 3–5) participants with PAE [30]. Another trial of choline in children with PAE who were 5 to 10 years old did not find beneficial effects on cognitive functioning compared to placebo, suggesting that a sensitive or critical period for choline effects on cognition occurs relatively early in postnatal life [33].

Here, we present a follow-up study of the child participants treated with choline or placebo in our first two trials to evaluate the potential long-term cognitive and behavioral implications of an early nutritional intervention targeting neurodevelopment. In addition to a global measure of cognition (Stanford–Binet Intelligence Scales), domain-specific outcomes focusing on memory, attention, executive functioning, and related behaviors were also measured. These domains were included in light of the pre-clinical evidence of choline-related improvements following PAE in visual-spatial learning [27], spatial reversal learning [26], and hyperactivity [36] as well as benefits in the hippocampus and pre-frontal cortex in rodent models [37].

## Materials and methods

### Parent-study methods and participants

Participants were children with PAE who took part in an earlier clinical trial of choline supplementation [30]. The initial study was a randomized, double-blind, placebo-controlled trial conducted at the University of Minnesota between June 2010 and May 2014. Participants underwent an IRB-approved informed consent process. Additional oversight was provided by the university’s clinical trial monitoring program as well as an independent Data Safety Monitoring Board. Choline was studied under the Federal Drug Administration (FDA) Investigational New Drug (IND) application #107085. The trial was registered with ClinicalTrials.Gov (#NCT01149538) on June 21, 2010, prior to the first participant’s enrollment. A complete description of methods and procedures was reported in Wozniak et al. [29]. Results of the initial trial were reported in Wozniak et al. [30].

**Fig. 1:**
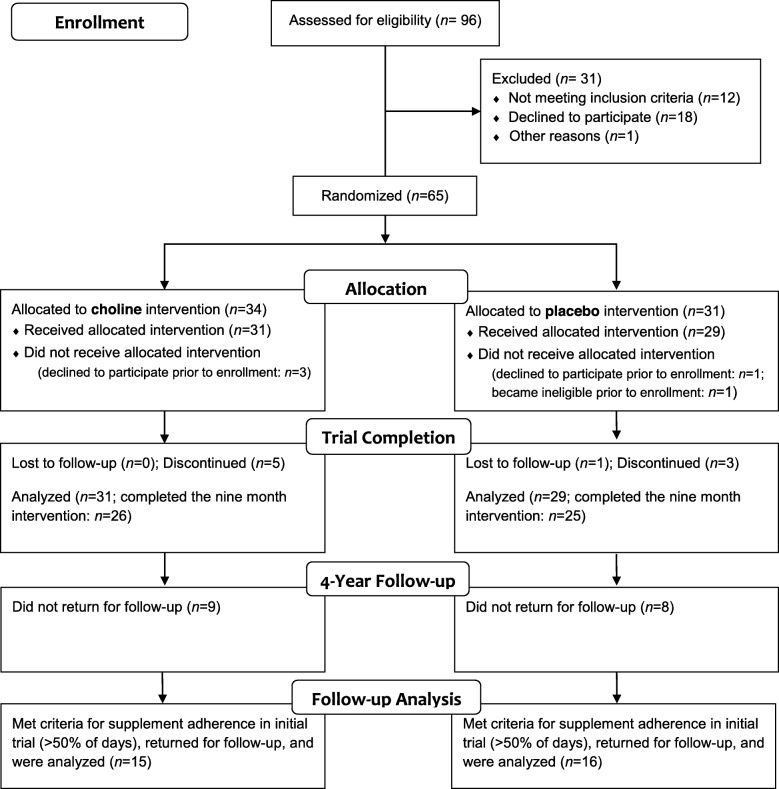
CONSORT flow diagram for both the initial randomized controlled trial and the 4-year follow-up study

Children with FASD (ages 2.5–5.0 years at enrollment) were initially recruited from the University’s FASD Clinic and Adoption Medicine Clinic. Sixty children received the allocated intervention of choline or placebo (1:1 allocation to parallel groups), of which 85% (*n* = 51) completed the 9-month trial (Fig. 1) [30]. The block randomization schedule was computer-generated by a statistician prior to the study and drug dispensing was handled by a university investigational drug services pharmacy, keeping the investigators, staff, and participants blind to allocation. The initial sample size was set for the detection of an effect size of 0.43 on the primary outcome (Mullen Scales of Early Learning). Initial exclusion criteria were the presence of another developmental disorder (e.g., autism, down syndrome), neurological disorder, traumatic brain injury, or other medical conditions affecting the brain. Psychiatric co-morbidity, such as attention deficit hyperactivity disorder or learning disorder, was not exclusionary as co-morbidity is common in FASD [38]. All but one participant, a twin born at 36 weeks weighing 1360 g, had a birthweight > 1500 g.

Between December 2015 and November 2017, participants who had completed the initial trial were asked to return for a long-term follow-up visit. A number of participants did not return for follow-up due to lost contact or no response to the invitation; 9 choline group participants (35%) and 8 placebo group participants (32%) did not return. Chi-square tests and *t* tests comparing non-returning participants to returning participants revealed no significant differences in age at enrollment, race, ethnicity, facial characteristics, height or weight deficiency, deficient brain growth, alcohol exposure confirmation, drug exposure, diagnostic category, or baseline Mullen Early Learning Composite. The characteristics of the returning sample are included in Table 2. At baseline, all participants were characterized according to the modified Institute of Medicine (IOM) criteria [39]. Of the returning participants, 4 (13%) had met criteria for FAS; 13 (42%) for pFAS; and 14 (45%) for ARND (Table 2). The average length of follow-up was 4.0 years from the completion of the trial. Height and weight, taken during the initial study, were standardized using normative data from the Centers for Disease Control [40]. Head circumference measures, also taken during the initial study, were characterized using World Health Organization normative data [41]. Overall, the returning participants were balanced across groups; there were no significant differences between returning participants from the choline group vs. the placebo group in terms of age at enrollment or age at follow-up, sex, racial or ethnic background, dysmorphology, growth, alcohol or other drug exposure, baseline cognitive functioning (Mullen Scales) or FASD diagnosis.

**Table 2:** Characteristics of participants

*N*(%) or mean (SD)	Placebo (*n* = 16)	Choline (*n* = 15)	Statistical test
Age at enrollment (years)	3.95 (0.75)	3.81 (0.83)	*t* (29) *= 0.51 p = 0.62*
Age at follow-up (years)	8.59(0.99)	8.57 (1.01)	*t* (29) = 0.04 *p* = 0.97
Years since study completion	3.94 (0.60)	4.03 (0.48)	*t* (29) = 0.44 *p* = 0.67
Gender			
Male	7 (43.8%)	8 (53.3%)	*χ*^*2*^ (1) = 0.29 *p* = 0.60
Female	9 (56.2%)	7 (46.7%)	–
Racial categories			
White	5 (31.3%)	8 (53.3%)	*χ*^*2*^ (5) = 0.40 *p* = 0.56
Black or African American	5 (31.3%)	2 (13.3%)	
American Indian/Alaska Native	3 (18.8%)	1 (6.7%)	
Asian	1 (6.3%)	1 (6.7%)	
More than one race	2 (12.5%)	2 (13.3%)	
Not reported	0 (0%)	1 (6.7%)	
Ethnic category			
Hispanic or Latino	1 (6.3%)	1 (6.7%)	*χ*^*2*^ (2) = 0.97 *p* = 0.62
Not Hispanic or Latino	14 (87.5%)	14 (93.3%)	
Unknown	1 (3.2%)	0 (0.0%)	
Dysmorphic facial features			
Lip (score 4 or 5)	11 (68.8%)	7 (50.0%)	*χ*^*2*^ (1) = 1.09 *p* = 0.30
Philtrum (score 4 or 5)	13 (81.3%)	7 (50.0%)	*χ*^*2*^ (1) = 3.28 *p* = 0.07
Palpebral fissure (≤ 10th percentile)	11 (68.8%)	11 (78.6%)	*χ*^*2*^ (1) = 0.37 *p* = 0.54
≥ 2 facial features present	11 (68.8%)	7 (46.7%)	*χ*^*2*^ (1) = 1.55 *p* = 0.21
Growth deficiency (≤ 10th percentile)			
Height	4 (25%)	4 (13.3%)	*χ*^*2*^ (1) = 0.68 *p* = 0.41
Weight	2 (12.5%)	4 (26.7%)	*χ*^*2*^ (1) = 1.00 *p* = 0.32
Deficient brain growth (≤ 10th percentile)			
Occipital-frontal circumference (OFC)	6 (37.5%)	5 (33.3%)	*χ*^*2*^ (1) = 0.06 *p* = 0.81
Alcohol exposure			
Alcohol confirmed	14 (87.5%)	12 (80.0%)	*χ*^*2*^ (1) = 0.32 *p* = 0.57
Alcohol suspected	2 (12.5%)	3 (20.0%)	
Drug exposure			
Other drug exposure suspected	8 (50.0%)	9 (60.0%)	*χ*^*2*^ (1) = 0.31 *p* = 0.58
IOM diagnostic category			
FAS	3 (18.8%)	1 (6.7%)	*χ*^*2*^ (2) = 1.33 *p* = 0.52
Partial FAS	7 (43.8%)	6 (40.0%)	
ARND	6 (37.5%)	8 (53.3%)	
Baseline Mullen Early Learning Composite	77.9 (22.0)	84.5 (13.4)	*t* (29)= − 0.98 *p* = 0.34

Details of the initial trial design have been published previously [30]. Briefly, participants were randomized (1:1) to choline (1.25 g. choline bitartrate powder delivering 513 mg choline) or placebo daily for 9 months. The study drug, delivered in a fruit-flavored powdered drink mix, was administered by the child’s parent(s). Compliance, as measured by calendar logs and packet counts, was equally high for both groups with study drug being consumed on 88% of the days in the study. Participants were assessed with a measure of global cognitive functioning—the Mullen Scales of Early Learning [42]—at baseline and again at the 9-month conclusion of the supplement period. An elicited imitation (EI) paradigm [43, 44] provided measures of hippocampus-dependent sequential memory at baseline, 6 months, and 9 months. The specific EI paradigm (described in [30]) involved demonstrating 9-step thematic sequences with toys and then allowing the child to imitate the sequences. For example, one sequence involved “going camping” and included steps such as setting up a toy tent, “baiting” a toy fishing pole with a magnet, and “catching” a toy fish. Children were asked to imitate the sequence after a 15-min delay. Scores from two different sequences were averaged. Parents also completed the Child Behavior Checklist (CBCL) [45] as a comprehensive report on the child’s behavior at baseline and at study completion.

### Follow-up methods

The 4-year follow-up was completed in one visit. Participants were included in the follow-up study if the child had received the study drug on at least 50% or greater of the days in the initial 9-month trial. Two participants in the choline group and one participant in the placebo group were dropped from the analyses because of poor compliance. Remaining participants were, on average, 8.6 years of age at follow-up and, therefore, the Mullen Scales were no longer age-appropriate.

#### General cognitive functioning

The Stanford–Binet Intelligence Scale, Fifth Edition (SB-5) [46] was administered as a measure of global cognitive functioning. The SB-5 yields a Full-Scale IQ score as well as standardized scale scores for verbal IQ, non-verbal IQ, fluid knowledge, quantitative reasoning, visual-spatial ability, and working memory skill. SB-5 scores have a mean of 100 and a standard deviation of 15.

#### Memory functioning

An EI paradigm, similar to that used in the initial trial, was employed using more difficult event sequences and a longer (60 to 80 min) memory delay to account for the older age of the participants at follow-up and to minimize potential ceiling effects. In the initial trial, the EI paradigm used event sequences that had high or medium levels of “connectivity” between steps in the sequence. For example in the camping scenario, several steps were logically connected or linked (one cannot “catch” a fish before “baiting” the fishing line). In the follow-up, steps in the event sequence were not logically connected (low connectivity), therefore requiring the child to rely strictly on the order in which the items were presented rather than having a natural “framework” to guide the memory for the sequences. For the follow-up, two different low-connectivity event sequences were used and the scores were averaged together. Sessions were video-recorded and scored offline by trained raters. Twenty percent of the videos were coded by two raters to ensure reliability (93%). EI scores include the percentage of individual steps recalled, percentage of ordered items recalled, and percentage of adjacent ordered pairs recalled correctly. In addition, three subtests from the NEPSY-II Developmental Neuropsychological Assessment [47] were administered: Memory for Names, Memory for Faces, and Narrative Memory. Memory for Names required the child to view 6–8 line drawings of faces and memorize the names associated with the faces over a series of learning trials with feedback. The recall is after a 25–35-min delay. Memory for Faces requires a child to view 16 black and white photographs of faces and, following a 15–25-min delay, identify the familiar faces that are presented alongside two distractors in three-choice arrays. Narrative Memory requires the child to listen to a short story and immediately recall as much detail as possible. Recognition cues are provided for missing details. NEPSY-II scores have a mean of 10 and a standard deviation of 3.

#### Executive functioning

Measures from the National Institutes of Health (NIH) Toolbox [48] were also administered electronically (iPad) at follow-up. Subtests administered were the Flanker Inhibitory Control and Attention Test, Dimensional Change Card Sort Test, and Picture Sequence Memory Test. NIH Toolbox *T* scores have a mean of 50 and a standard deviation of 10.

#### Behavioral and emotional functioning

A parent completed the Child Behavior Checklist (CBCL) at follow-up. From the CBCL, only the attention deficit hyperactivity (ADHD) problems scale was analyzed—an a priori decision based on the hypothesis that changes in memory and executive functioning would potentially be reflected in ADHD-related behaviors. CBCL *T* scores have a mean of 50 and a standard deviation of 10.

### Statistical analyses

Statistical analyses were conducted with IBM SPSS Version 25. Analyses are described together with their results. The number of analyses was intentionally limited because of the small sample size and no additional measures were implemented to correct for multiple testing.

## Results

### Participant characteristics

Returning participants from the choline and placebo groups were well-matched and not significantly different in age, gender, race, ethnicity, FASD diagnostic criteria, alcohol and other drug exposure, or resulting FASD diagnoses (Table 2).

### General cognitive functioning

Scores from the SB-5 at follow-up were compared across groups (choline vs. placebo) with a univariate General Linear Model (GLM) analysis for Full-Scale IQ and a separate multivariate GLM for the index-scores: Verbal IQ, Non-Verbal IQ, Fluid Reasoning, Knowledge, Quantitative Reasoning, Visual-Spatial Processing, and Working Memory. Both models included the participant’s Mullen Scales of Early Learning Composite Score from the baseline visit in the trial (administered approximately 4 years prior on average) as a covariate. The univariate analysis revealed no Full-Scale IQ difference. The multivariate model revealed a significant group effect (choline vs. placebo), Wilk’s Lambda = 0.52, *F* (7, 20) = 2.62, *p* = 0.04. Table 3 contains the results for each of the indices and the Full-Scale IQ scores. There were two significant group differences: non-verbal IQ was 8.2% higher for those who had received choline vs. those who had received placebo, and those in the choline group had working memory scores that were 11.7% higher than those in the placebo group.

**Table 3:** Stanford–Binet Intelligence Scale—Fifth Edition group comparison results

EMMean (SE)	Placebo (*n* = 16)	Choline (*n* = 14)^a^	Statistic	Significance	Effect size
Verbal IQ	88.3 (2.8)	90.6 (3.1)	*F* (1, 28) = 0.29	*p* = 0.60	PE^2^ = 0.01
Non-Verbal IQ	85.6 (2.1)	92.9 (2.4)	*F* (1, 28) = 5.17	*p* = 0.03*	PE^2^ = 0.17
Fluid Reasoning	88.1 (3.7)	90.3 (4.1)	*F* (1, 28) = 0.15	*p* = 0.70	PE^2^ = 0.01
Knowledge	85.0 (2.3)	87.5 (2.6)	*F* (1, 28) = 0.50	*p* = 0.49	PE^2^ = 0.02
Quantitative Reasoning	93.1 (2.1)	92.7 (2.3)	*F* (1, 28) = 0.02	*p* = 0.90	PE^2^ = 0.00
Visual-Spatial Processing	91.3 (3.0)	98.3 (3.3)	*F* (1, 28) = 2.38	*p* = 0.14	PE^2^ = 0.08
Working Memory	84.0 (2.5)	94.4 (2.8)	*F* (1, 28) = 7.74	*p* = 0.01*	PE^2^ = 0.23
Full-Scale IQ	86.1 (2.4)	91.1 (2.7)	*F* (1, 28) = 1.86	*p* = 0.19	

Note: All analyses controlled for baseline cognitive functioning by including the participant’s Mullen Scales of Early Learning Composite score as a covariate

*significance *p* <.05

*EMMean* estimated marginal means, *SE* standard error, *PE*^2^ partial eta^2^ values

^a^Stanford–Binet scores were not obtained from two participants in this group due to time constraints

Because the non-verbal IQ is comprised of five components (non-verbal Fluid Reasoning, non-verbal Knowledge, non-verbal Quantitative Reasoning, non-verbal Visual-Spatial Reasoning, and non-verbal Working Memory, we conducted post hoc GLMs to determine which of the five component(s) contributed to the overall beneficial group effect for choline on Non-Verbal IQ. Of these five non-verbal components, there were significant group effects of choline for the non-verbal Visual-Spatial Reasoning component (mean = 10.98 for choline and mean = 8.20 for placebo; a 28.9% difference), [*F* (1, 29) =9.93, *p* = .004] and the non-verbal Working Memory component (mean = 9.08 for choline and mean = 6.93 for placebo; a 26.8% difference), [*F* (1, 29) = 6.37, *p* = .018] but not the other three components. Similarly, we conducted additional analyses to determine how the two components of Working Memory (verbal Working Memory and non-verbal Working Memory) contributed to the overall finding of improved Working Memory for the choline group. The analysis of verbal Working Memory did not reveal a significant effect [*F* (1, 29)=2.73, *p* = .110] but the non-verbal Working Memory component did (see above). In summary, choline had had specific effects on the non-verbal aspects of working memory and on visual-spatial processing but non-significant effects on quantitative reasoning, fluid reasoning, and a range of verbally-mediated skills.

### Memory functioning

Three EI measures (short-delay components, pairs, and adjacent pairs) at follow-up were compared across groups (choline vs. placebo) with univariate GLM analyses, each controlling for the comparable EI score from baseline (components, pairs, and adjacent pairs). As shown in Table 4, there were no significant differences at follow-up. Because the initial study found that age at enrollment moderated the effect of choline vs. placebo on EI performance, potential moderating effects of age on the long-term follow-up EI performance were tested with GLM analyses. Age was not a significant moderator for EI short-delay components [*F* (1, 27) = 1.63, *p* = 0.21], EI pairs [*F* (1, 27) = 1.76, *p* = 0.20], or EI adjacent pairs [*F* (1, 27) = 1.61, *p* = 0.22]. For the NEPSY-II memory subtests administered at follow-up, there was no equivalent test administered at baseline; therefore, univariate analyses were conducted without covariates. As shown in Table 4, participants in the choline group had significantly higher Memory for Names Delayed scores compared to those in the placebo group (37.9% difference, Cohen’s *d* = 0.77).

**Table 4:** Memory and executive functioning group comparison results

Mean (SE) {*n*}	Placebo	Choline	Statistic	Sig.	Effect size
*Memory*					
EI short delay components ^a^	96.1(0.9) {16}	97.8 (0.9) {15}	*F* (1, 30) = 1.89	*p* = 0.18	PE^2^ = 0.06
EI short delay pairs ^a^	63.2 (2.6) {16}	63.8 (2.7) {15}	*F* (1, 30) = 0.29	*p* = 0.87	PE^2^ = 0.00
EI short delay adjacent pairs ^a^	21.3 (3.8) {16}	24.7 (4.0) {15}	*F* (1, 30) = 0.38	*p* = 0.55	PE^2^ = 0.13
NEPSY-II Memory for Names Delayed	6.2 (3.2) {16}	9.1 (4.3) {15}	*F* (1, 30) = 4.75	*p* = 0.04*	*d* = 0.77
NEPSY-II Memory for Faces Delayed	8.8 (3.7) {13}	8.7(2.3) {14}	*F* (1, 26) = 0.00	*p* = 0.96	*d* = 0.03
NEPSY-II Narrative Memory	7.8 (3.6) {16}	8.3 (2.4) {15}	*F* (1, 30) = 0.01	*p* = 0.93	*d* = 0.16
NIH Toolbox PSMT	47.4 (13.6) {16}	50.9 (14.2) {15}	*F* (1, 30) = 0.49	*p* = 0.49	*d* = 0.25
*Executive functioning*					
NIH Toolbox DCCST	40.4 (7.5) {15}	44.1 (10.8) {15}	*F* (1, 29) = 1.29	*p* = 0.27	*d* = 0.40
NIH Toolbox Flanker test	39.8 (8.0) {16}	45.6 (9.6) {15}	*F* (1, 30) = 3.32	*p* = 0.08	*d* = 0.66

Note: *NEPSY-II* Narrative Memory score provided is the free and cured recall total score

*significance *p* <.05

*EI* elicited imitation, *SE* standard error, *PE*^2^ partial eta squared, *d* Cohen’s *d* effect size, *DCCST* Dimensional Change Card Sort Test, *PSMT* Picture Sequence Memory Test

Note: EI analyses controlled for baseline EI score (entered as covariates)

^a^ Estimated marginal means are provided

### Executive functioning

Univariate GLM analyses without covariates were conducted for the two executive functioning subtests from the NIH Toolbox. There was no group difference for the Dimensional Change Card Sort Test. For the Flanker Inhibitory Control Test, there was a trend (*p* = 0.08) toward higher performance in the choline group compared to the placebo group **(**Table 4**)**. Although the Flanker task group difference was not statistically significant, the effect size was moderate (*d* = 0.66), representing a 13.5% difference.

### Behavioral and emotional functioning

A univariate analysis of the CBCL parent-reported ADHD problems scale at follow-up was conducted with the equivalent score from baseline entered as a covariate. Participants who had received choline had significantly lower scores on this scale (estimated marginal mean = 62.1; SE = 2.1) compared to the placebo group (estimated marginal mean = 69.0; SD = 2.0), *F* (1, 28) = 5.57, *p* = .026, partial eta squared = 0.17. This difference represents a 10.5% difference between the groups with the choline group showing fewer ADHD-related behavioral problems than the placebo group at follow-up.

## Discussion

The data presented here demonstrate significant long-term neurodevelopmental benefits 4 years after choline supplementation in children with FASD. A noteworthy aspect of these findings is that the long-term effect sizes are larger and more consistent across measures than those observed immediately following the trial completion. Specifically, the initial treatment effects were only apparent in delayed sequential memory in only the younger group of children (ages 2 and 3) and were not observed for any of the intelligence scales (Mullen Early Learning Scales) [30]. At 4-year follow-up, the treatment effects were broader and included significant differences in intelligence test scale scores (SB-5), delayed verbal memory (NEPSY-II memory for names), and ADHD-related behavioral problems (CBCL). At the initial completion of the trial, effect sizes for the EI memory paradigm in the younger children were “medium” (Cohen’s *d* values of 0.54 and 0.50, respectively, for short-delay components and ordered pairs), whereas at the 4-year follow-up, the effect sizes were “medium” to “large” (partial-eta^2^ = 0.17, Cohen’s *f* = 0.38 for non-verbal IQ and partial-eta^2^ = 0.23, Cohen’s *f* = 0.43 for working memory; In addition, a medium to large effect size (Cohen’s *d* = 0.77) was observed for delayed verbal memory (NEPSY-II memory for names). For reference, Cohen’s *f* effect size descriptors are small = 0.1; medium = 0.25; and large = 0.40 (from Cohen’s original text [49]).

It is worth noting that the effects of choline on declarative memory functioning observed here are consistent with findings from two previous studies of choline supplementation in FASD. In one prenatal supplementation study, Kable et al. administered choline + multivitamin vs multivitamin alone to Ukrainian women who drank heavily during pregnancy [31]. They observed improvements in basic information processing in the 6-month-old infants who had received choline during gestation as measured by a cardiac-orienting response task. Infants who received choline showed different cardiac orienting responses (larger changes in heart rate, shorter latency of response, etc.), suggesting that they had encoded the visual stimuli (faces) in memory and recognized the novel stimuli (unfamiliar faces) more readily than infants who did not receive choline. That study found no effects of choline on auditory processing, however. In the second study of prenatal choline supplementation, Jacobson et al. administered choline vs. placebo to South African women who drank heavily during pregnancy and studied the offspring at 6 and 12 months of age [34]. At 12 months, infants who had received choline during gestation performed better on the Fagan Test of Infant Intelligence, showing a preference for novel stimuli which indicates better visual recognition memory. Taken together with these prior results, the current findings of improved visual-sequential memory and verbal declarative memory for the choline group strongly suggest that recognition and retrieval should continue to be domains of interest in future studies of choline supplementation in FASD.

The results presented here highlight both the challenges and importance of measuring specific treatment effects longitudinally following a neurodevelopmental intervention. Measuring the neurodevelopmental effects of choline supplementation in FASD is challenging given the heterogeneity of the underlying brain insults that result from PAE. Alcohol’s effects on the developing brain are widespread [50–53] and vary across individuals due to the range of doses (drinking patterns, blood alcohol levels reached), differential exposure patterns (early cessation vs. first trimester vs. throughout gestation), and numerous individual interacting factors (genetic, maternal diet, comorbid substance use) [54]. In contrast to alcohol’s widespread damaging effects on critical early neurodevelopment, postnatal interventions, such as nutritional supplementation, are likely to have limited and specific neural targets and will only address remaining neurodevelopment going forward from that point. Therefore, in these types of postnatal trials, small incremental cognitive benefits for some individuals in select cognitive domains, similar to those observed in the initial choline supplementation trial, [30] are expected rather than large-scale, global cognitive, and behavioral improvements. The current finding that choline impacted non-verbal working memory more than verbal working memory is an example of the type of specific benefit that might be expected.

It is also critical to recognize that neurodevelopment is a protracted process in which early insults such as PAE play out over time and are not fully captured with a single measurement point. As an example, rodent models of PAE show reductions in cortical volume and surface area that become more apparent during later adolescence—a period during which cortical volume and surface normally increase in non-exposed animals [55]. Similarly, a longitudinal MRI study of children with FASD revealed alterations in the typical developmental trajectory of cortical volume [56], a finding that was only apparent when examining the course of development over multiple time points. The current findings are consistent with the hypothesis that downstream benefits of early neurodevelopmental interventions may only become apparent after longer periods of follow-up. A similar pattern was observed in a study of long-chain polyunsaturated fatty acid (LCPUFA) supplementation in typically developing infants in which the beneficial effects for cognitive functioning were not seen initially (18 months of age) but did manifest at the 3-year to 6-year assessment points [57].

The current data reveal that, in addition to beneficial cognitive effects, choline supplementation was also associated with parent-reported improvements in attention and behavioral regulation in children at 4-year follow-up (CBCL ADHD scale). This finding is important because it is well-established that behavioral disorders commonly co-occur with FASD, likely as a result of both the direct impact of PAE on neural systems involved in impulse control, behavior regulation, and judgment as well as the combined effects of poverty, neglect, abuse, other substance abuse, and parental mental health difficulties. FASD meta-analyses demonstrate very significant increases in behavioral disturbance (e.g., ADHD: 8 to 10-fold increase over population prevalence rates in the literature and published by the US National Institutes of Health) and language disorder (10-fold) [58, 59]. The mechanism by which early choline supplementation could influence later ADHD-related behavior is not known but could be partly a function of improvements in core cognitive skills including working memory, learning, and non-verbal reasoning. Supplementation may also enhance the early development of neural circuits that become important in higher-level cognitive functioning at later stages of development as has been shown with iron supplementation for iron deficiency in animal models [60, 61].

The neurobiological function of choline in development may have at least three underlying components: the production of cell membrane phospholipids for axonal growth and myelination, enhancement of available acetylcholine, and epigenetic effects related to DNA methylation [20]. First, choline is required for the production of phosphatidylcholine, sphingomyelin, and plasmalogens—lipids that are present in all cell membranes. In neurons, these membrane phospholipids are necessary for axonal growth and myelination, among other processes [13]. Second, the ability of cholinergic neurons to produce acetylcholine is directly related to the availability of free choline—its precursor [62]. Choline also affects choline acetyltransferase levels in the hippocampus and frontal cortex in rats and is associated with improved memory functioning, especially visual-spatial memory [21, 22]. In animal models, choline contributes to increased hippocampal dendritic arborization in CA1, larger cells, and functional changes to the cells [14–17]. Postnatal choline may enhance synaptogenesis and ongoing hippocampal growth [20], which is known to proceed rapidly during the first 2 years of life and more slowly after [63]. In humans, the hippocampus continues to develop into the fourth year [64]. Third, choline provides methyl-donor groups that facilitate DNA methylation which, ultimately, plays a role in gene expression [13, 65]. In rodents, choline reduces the excessive methylation following PAE [37]. Using blood samples from participants in our initial choline trial, we recently demonstrated that choline supplementation reduced DNA methylation and increased expression of two stress regulatory genes (PER2 and POMC) in children with FASD [35].

Some limitations should be considered in placing the results of the current study in the context of the existing literature. First, the challenges of identifying and enrolling pre-school age children with PAE and following them over the course of several years of development contributed to relatively small sample size for this study. Although this tempers the conclusions that can be drawn from the data, it is noteworthy that significant effects for choline supplementation were able to be detected even with a small sample size. Larger studies in the future may allow for multiple doses and/or different lengths of supplementation to be tested directly against each other—which will be important in moving toward broader treatment implementation. A second limitation to consider is that the randomized controlled trial design utilized here did not include a non-alcohol-exposed group. Therefore, it is not entirely clear whether choline would have developmental benefits of the type measured here even in children without the effects of PAE. We are not aware of any published studies that administered choline to typically developing pre-school children and evaluated cognitive outcome. One prenatal study of healthy pregnancies found no benefits of second and third-trimester maternal choline supplementation on the offspring’s cognitive functioning [66]. However, another prenatal supplementation study did reveal a dose-dependent beneficial effect of third-trimester maternal choline supplementation on the offspring’s information processing [67]. That study found that the choline group showed faster saccadic reaction times on a visual task, and the authors noted that saccadic reaction time is known to be predictive of cognitive functioning later in typical development. A third limitation of the current study is that we do not know whether choline supplementation was beneficial because it corrected a deficiency in the children’s diets or whether it would be beneficial regardless of dietary intake. A snapshot of dietary intake from our previous studies does suggest that preschool-age children with FASD have high rates of dietary insufficiency for choline and other micronutrients [68], possibly because of taste and texture sensitivities and abnormal eating behaviors [69]. Future studies of choline supplementation in children with PAE may benefit from detailed analyses of children’s dietary intake as a potential mediator of the outcome. Future studies might also test actual dietary interventions as a more natural method of achieving nutritional sufficiency compared to single-nutrient supplementation.

## Conclusions

Realistically, nutritional interventions addressing neurodevelopmental disorders need to mesh with a host of other interventions and accommodations to meet a child’s specific profile of behavioral, mental health, cognitive, adaptive, and social needs [8, 70]. Ultimately, for children and adolescents with FASD, interventions may include a combination of components including nutritional supplementation, parent-education and behavior management training, computerized attentional training, impulse control therapy, special education including literacy and math training, and social skills development [8]. These components will likely be differentially effective depending on individual cognitive profiles and depending on the developmental windows during which they are administered. Beyond individual interventions, at the public health level, it is critical to continue to address FASD through robust support of addiction treatment, alcohol abstinence, birth control, and public awareness that there is no safe level of alcohol consumption during pregnancy [71, 72].

## Data Availability

The datasets used and/or analyzed during the current study are available from the corresponding author on reasonable request. Outcome data from the parent trial is available on ClinicalTrials.Gov.

## Abbreviations

ARND: Alcohol-related neurodevelopmental disorder
FAS: Fetal alcohol syndrome
FASD: Fetal alcohol spectrum disorders
pFAS: Partial fetal alcohol syndrome
